# Cell-Based Modeling of Tissue Developing in the Scaffold Pores of Varying Cross-Sections

**DOI:** 10.3390/biomimetics8080562

**Published:** 2023-11-21

**Authors:** Ivan Krasnyakov, Dmitry Bratsun

**Affiliations:** Applied Physics Department, Perm National Research Polytechnic University, 614990 Perm, Russia; krasnyakov_ivan@pstu.ru

**Keywords:** tissue engineering, vertex model, scaffold occupation, shear stress effect

## Abstract

In this work, we present a mathematical model of cell growth in the pores of a perfusion bioreactor through which a nutrient solution is pumped. We have developed a 2-D vertex model that allows us to reproduce the microscopic dynamics of the microenvironment of cells and describe the occupation of the pore space with cells. In this model, each cell is represented by a polygon; the number of vertices and shapes may change over time. The model includes mitotic cell division and intercalation. We study the impact of two factors on cell growth. On the one hand, we consider a channel of variable cross-section, which models a scaffold with a porosity gradient. On the other hand, a cluster of cells grows under the influence of a nutrient solution flow, which establishes a non-uniform distribution of shear stresses in the pore space. We present the results of numerical simulation of the tissue growth in a wavy channel. The model allows us to obtain complete microscopic information that includes the dynamics of intracellular pressure, the local elastic energy, and the characteristics of cell populations. As we showed, in a functional-graded scaffold, the distribution of the shear stresses in the pore space has a complicated structure, which implies the possibility of controlling the growth zones by varying the pore geometry.

## 1. Introduction

In recent years, tissue engineering has become one of the leading research areas in the biomedical sciences. Bioartificial organ manufacturing technologies are a series of enabling techniques used to produce human organs based on bionic principles. The development of these technologies holds the promise to radically improve the quality of health and the average lifespan of human beings in the near future [1,2]. As is known, any tissue is a multicellular structure. Under natural conditions, a tissue develops in the process of morphogenesis. Hundreds and possibly thousands of genes control this process. Their sequential expression triggers a system of electrical, chemical, and mechanical signals [3]. This process leads to the local differentiations of cells, which form tissues of different functionalities. This implies that tissue engineering must learn how to launch the in vitro process, which mimics natural morphogenesis. In fact, the development of these technologies is at the beginning of the path due to the complexity of the problem. At the moment, tissue engineering can synthesize relatively simple and homogeneous cell aggregates. The book [4] reviews recent developments in this area.

At the moment, tissue scaffold represents a principal component for tissue engineering [2,5]. It provides a suitable mechanical and chemical environment for cell growth and differentiation. The scaffold matrix can be either porous or fibrous material. This material is then filled with a nutrient solution and seeded with undifferentiated cells. If the scaffold is part of a perfusion bioreactor, then the growing tissue represents a dynamic cell culture. Otherwise, the final product is a static culture [6].

It is important to note that hydrodynamic phenomena play a crucial role in tissue growth since shear stresses stimulate cells to divide [7,8]. Many studies show that the rate of tissue growth can increase several times, but the effect of stresses is not linear [9,10]. The intensity of proliferation can increase five times [11]. However, at high stresses, cells begin to die. Thus, the effect manifests itself in a resonant manner. Some authors indicate the most comfortable shear stress value is $5\times 10^{-5}$ Pa [7]. Cells die at stresses higher than $50\times 10^{-3}$ Pa [12]. Thus, the flow in the perfusion bioreactor should not generate stress higher than $5\times 10^{-3}$ Pa [13].

In tissue engineering, researchers usually seek to create the most efficient scaffold topology, providing the highest tissue growth rate [14]. However, they encounter a variety of configurations when selecting scaffolds for tissue engineering. Presumably, scaffolds should imitate the extracellular matrix of native tissue, allowing cells to establish their microenvironment and thus reproduce the structure of the future tissue, at least approximately. It is important to remember that a solid matrix that comes into contact with the cells in the scaffold is not a natural neighbor of the cells. All the details of the interaction between cells and solid surfaces are still unknown. For example, experimental observations using confocal microscopy differentiate two types of cell attachment in the collagen fibrous scaffolds: about 25% of the cells are attached flatly to the solid surface, but most of them are oriented such that they transversely bridge two struts [15,16]. More recent studies confirmed these findings [17,18,19]. In the case of the porous scaffold, the cells prefer to lie flatly, covering the walls if the channel width is large enough [14]. Perhaps they perceive the walls of the pores as a basement membrane. Cell division leads to the filling of pores, while the cells grow both along the channel and towards its center, creating a multilayer structure.

**Figure 1:**
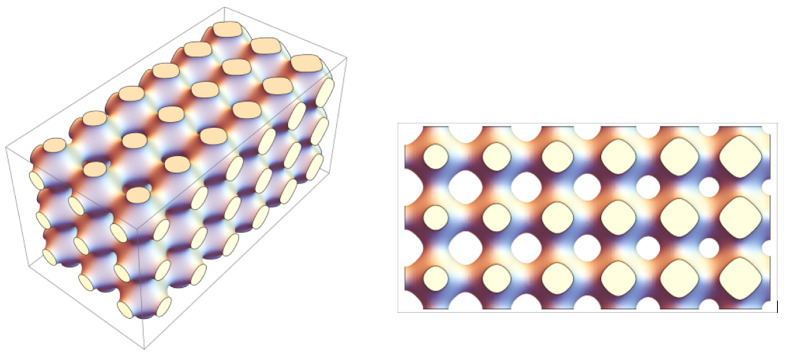
The scaffold defined by the Schwarz-R surface is a typical example of an artificial functionally graded material that is biomimetic of natural bone tissue [20,21,23].

So far, most authors have considered scaffolds with a spatially homogeneous structure. However, heterogeneous tissues cannot be grown on such a scaffold [20,21,22,23,24]. A matrix with a non-uniform porosity distribution is one of the ways to solve this problem (Figure 1). On the one hand, functionally graded materials are biomimetics of natural tissues [25,26], such as bone tissue. On the other hand, the manipulations with the internal structure of the scaffold make it possible to control the conditions for tissue growth. In particular, the variations in the spatial permeability of a solid matrix make it possible to change the shear stresses on walls and in a liquid. The latter, as noted above, has a direct effect on the rate of tissue growth. One could program the topology of the scaffold in advance in such a way as to structure the heterogeneity of the growing tissue. This technique opens up new development perspectives for tissue engineering.

Let us briefly discuss the main approaches to the mathematical modeling of tissue growth. The goal of modeling here is to analyze the biomechanical behavior of a cell cluster inside a scaffold [14]. The study of nutrient transport in pores is another stimulus for modeling. It enables the prediction of the distribution of the nutrient solution inside the scaffold and the evaluation of the efficiency of nutrient delivery to the cells [6,27]. Most works use the continuum approximation. These mathematical models represent the tissue as a continuous medium with a single set of properties [6,22,27]. Because of the complexity of the processes in the scaffolds, which simultaneously include mechanical, chemical, hydrodynamic, and biological interactions, such models require additional phenomenological assumptions. In the case of static cell culture, one commonly uses population dynamics models based on reaction–diffusion equations [22,28,29,30]. The models of the dynamic culture typically include space-averaged Darcy or Brinkman equations describing fluid flow through a porous medium [6,31,32,33,34]. The key disadvantage of these approaches is the lack of information about the dynamics of the cell microenvironment and some uncertainty of the parameter values used to describe the phenomenologically described continuum [14]. To correct these shortcomings, hybrid models were developed [35].

Discrete mathematical models of tissue growth represent an approach accounting for the microscopic processes in cells. Early attempts at such modeling were associated with cellular automata [36]. A cellular automaton typically consists of a regular grid of fixed elements (“cells”), each in one of a finite number of states. The system evolves according to some fixed rules. The individuality of the “cells” is not preserved in this approach. This approach is still popular due to its simplicity and robustness [37,38]. The further development led to the emergence of particle-based models [39,40,41], which are a variety of molecular dynamics models. With this approach, tissue elements are mobile, but the interaction of cells still does not look realistic. Finally, vertex models represent the most advanced approach to modeling a cell environment [42]. The key to the success and growing popularity of vertex models lies in the complex description of the structural unit of the environment. Each cell is represented here by a polygon. It can move in space and change its characteristics, keeping information about its individuality in the cellular ensemble [43,44,45]. Most models proposed in the literature are 2-D and focus on the tissue behavior along the apical surface. However, 3-D vertex models have also been developed (see, for example, [46]).

One of the advantages of vertex models is the ability to describe the diversity of cell behavior and their heterogeneity. They allow dynamic changes to be considered in cell properties [45]. Tissue growth models can take into account many details, including the mechanisms of gene regulation [47,48,49], the differentiation of cells according to their positions in the tissue [45], the variability in cell-to-cell interactions [50], the interaction of cells with the solid surface of the scaffold matrix [51], signaling pathways [34], and the influence of environmental factors [14]. However, vertex models also have their drawbacks. The requirements for computing resources increase rapidly with the number of cells, limiting the spatial and temporal scales of the systems under consideration. The most recent versions of the numerical implementation consider ensembles of several million cells, which are insufficient for modeling the tissue of even a small organ. Also, the interpretation of the simulation results can be non-trivial due to the complexity of the data. Finally, the discrete mathematical models of tissue growth represent a powerful tool for scaffold optimization. They allow a more detailed study of tissue development at the microscopic level, which encourages the emergence of new approaches in tissue engineering.

In this work, we study the effect of near-wall shear stresses that usually arise due to the adhesion of the pumped liquid to the channel surface, which leads to a difference in the fluid velocity. The shear stress field depends on the intensity of fluid flow, channel geometry, and solution viscosity. The first factor increases the velocity difference between two points in the channel. The second and third factors lead to a redistribution of the velocity field in the channel, creating stagnant zones or areas of high-speed fluid movement. If the cell size is comparable to the characteristic change in the velocity field, the cell experiences a stretching effect, stimulating the mitosis process. We develop a simple microscopic model of the 2-D tissue occupying the scaffold micropore. The vertex model includes the tissue interaction with the nutrient fluid flow in a dynamic culture developing in the perfusion bioreactor. We consider the case of a pore with a variable cross section being part of a functional-graded scaffold mimicking the structure of living bone tissue. The cell biomechanics model is coupled with the hydrodynamics of flow through a shear stress field, which stimulates cells to divide. The paper is organized as follows: In Section 2, we formulate the problem and discuss all aspects of the mathematical model. In Section 3, we present numerical results. Finally, Section 4 summarizes the results and provides some discussion.

## 2. Mathematical Formulation

### 2.1. Two-Dimensional Vertex Model for Simulating Tissue Growth

In this work, we study cell growth in the scaffold channel within the framework of a microscopic description. Cells divide and move under the influence of the crowding effect in the limited space of the pore. Also, the process proceeds under the significant impact of the shear stresses from the nutrient liquid flowing through the channel. If conditions for proliferation are favorable, cells form large dense clusters that resemble the packaging of natural tissues of living organisms.

A vertex model approach designed to describe dense cellular assemblies in the body is well suited to apply this problem. Generally, the process of tissue growth in the porous scaffold of some topology is three-dimensional. However, cells typically find a surface to which they tend to attach flatly. Under natural conditions, the basement membrane works as a supporting surface. If the characteristic pore size significantly exceeds the cell size, most of the cells in the scaffold prefer to attach flatly to the solid surface of the pore. Also, we should remember that the flow of the nutrient solution through the pores in a dynamic culture prevents cell attachment across the channel. The cell-division process results in the gradual filling of the pore, with cells growing along the pore surface, creating a dense structure (see, for example, experimental observations in [52]).

In their review [42], the authors made a helpful classification of vertex models. They noticed that 3-D models are still laborious to develop. Examples of the use of such models remain infrequent and are used to study systems with a small number of cells. Also, most problems can be reduced to 2-D vertex models, depending on what forces prevail in the tissue. If apical forces predominate, one can develop a 2-D apical model. Conversely, if lateral forces predominate, one can apply a 2-D lateral model [42].

**Figure 2:**
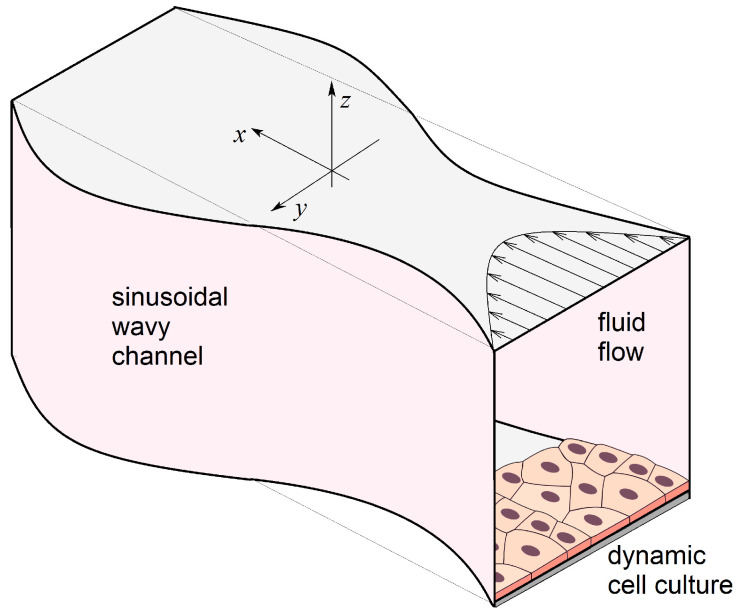
Schematic 3-D representation of a sinusoidal wavy channel with a flowing nutrient solution and a dynamic cell culture growing at the bottom.

Figure 2 schematically shows the configuration of a 3-D channel we consider in this work. The channel has a rectangular cross-section. The cells grow at the bottom of the cavity. The channel width varies harmonically along the *y*-axis but remains constant in the *z* direction. Rectangular channels often occur when using 3-D printers that print in a layer-by-layer mode. Similar structures arise when constructing scaffolds using struts [52].

With flat attachment, the stresses arising in the cell cytoskeleton are distributed unevenly. From general considerations, it is clear that peak stresses manifest themselves in the direction along the pore where the liquid flows. The effect of crowding cells perpendicular to a sidewall also generates significant pressure. In our problem, the tensile forces acting on cells in the *x*- and *y*-directions are an order of magnitude greater than the forces in the *z*-direction. Therefore, we can simplify the problem by considering only the apical cross-section of tissue. These considerations allow us to develop a quasi-2-D model. Additionally, in this work, we focus on cell-to-cell interactions that are weakly dependent on the global 3-D topology of the scaffold. Thus, we propose a 2-D apical vertex model, which looks sufficient to study the microscopic biomechanics of cells in the porous scaffold.

The basic idea of the vertex model is to represent a cell as a geometric object that is complex enough to carry individual traits and is capable of evolution. Typically, in 2-D vertex models, a cell is represented formally as a polygon. For simplicity, we will assume that all internal angles of a polygon at any time cannot exceed 180 degrees. Cells of the mesenchymal phenotype can take on very diverse forms due to their flexible cytoskeleton [53]. However, cells in dense clusters, not to mention natural tissues, are limited in their movements and belong to a passive phenotype. So we assume that the cells are tightly adjacent to each other, forming a continuous medium without gaps, as in natural tissue. With this packaging, cells are limited in their movements and can directly exchange mechanical and chemical signals with each other.

Let us discuss the main features of the developed mathematical model. To simulate the evolution of an ensemble of cells, we set the time-dependent elastic potential energy of the entire system:

(1) $$E\left(t\right)=\sum_{i=1}^{N(t)}E_{i}\left(t\right),$$

where

(2) $$E_{i}\left(t\right)=\frac{1}{2}\left[\mu {\left(L_{i}\left(t\right)-L_{0}\right)}^{2}+\eta {\left(A_{i}\left(t\right)-A_{0}\right)}^{2}\right].$$

**Figure 3:**
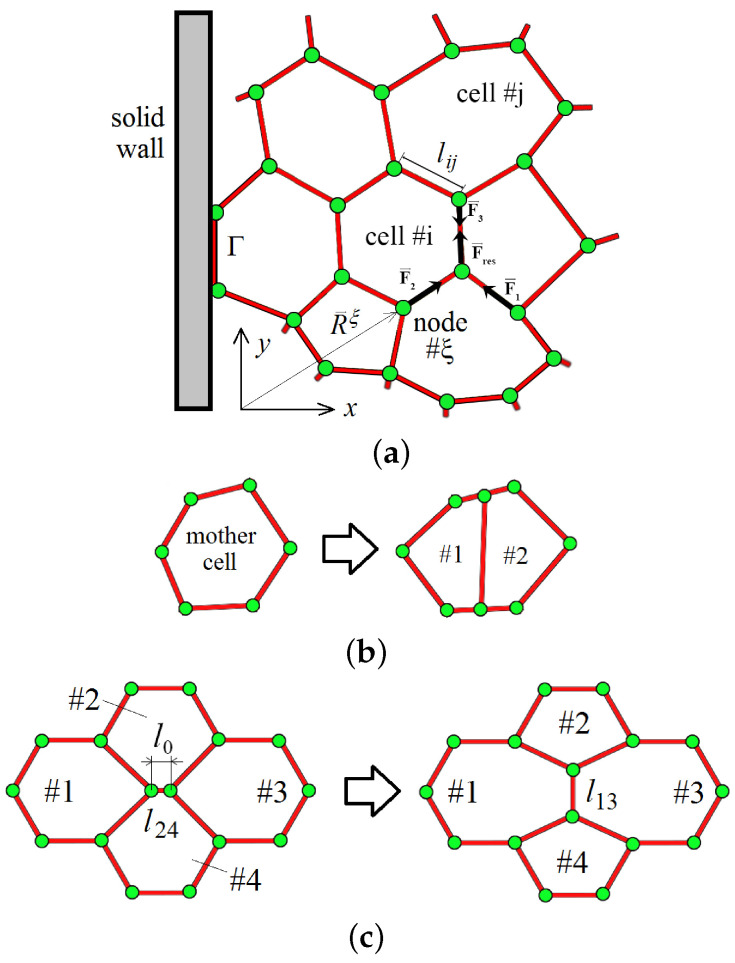
(**a**) The tissue in a scaffold pore is represented by an elastic 2-D array of polygonal cells tightly adjacent to each other. The green dots mark the vertices of polygons where forces (black arrows) are applied. The ability of cells to divide (**b**) and intercalate (**c**) makes the growing tissue mobile and fluidic.

Here, $L_{i}\left(t\right)$ and $A_{i}\left(t\right)$ represent the perimeter and area (“volume”) of the *i*-th cell at time *t*, respectively. Let us denote indices for numbering cells and nodes in Latin and Greek letters, respectively. The first term in Equation (2) describes the impact of the forces striving to reduce the perimeter difference of each cell to a minimum, and the second term expresses the cell’s resistance to stretching and compression forces as a cell attempts to preserve its reference area $A_{0}$. In (1), the summation is performed over all N(t) cells in the cluster. N(t) is a time-dependent function since the tissue grows. The perimeter of the *i*-th cell at time *t* is calculated as the sum of the sides of the polygon (Figure 3a):

(3) $$L_{i}\left(t\right)=\sum_{j=1}^{N_{i}\left(t\right)}l_{ij}\left(t\right).$$

The elasticity coefficients μ and η are the crucial parameters of the model that determine the deformation properties of the medium. The reference perimeter $L_{0}$ and area $A_{0}$ are the input parameters of the problem and are specified for a hexagonal-shaped abstract cell with a diameter *a* as follows:

(4) $$L_{0}=3a,\;A_{0}=\frac{3\sqrt{3}}{8}a^{2}=\frac{\sqrt{3}}{24}L_{0}^{2}.$$

Each polygonal cell changes its position in space by displacing its nodes (green circles in Figure 3). The resultant mechanical forces (black arrows) applied to each node are determined in a manner standard for the mechanics of conservative systems:

(5) $$F^{\xi }=-\frac{\partial E(t)}{\partial R^{\xi }},$$

where $R^{\xi }$ is the position vector of the ξ-th node. The displacement of nodes results in the deformation of the cell, changing its area and perimeter.

Let us discuss the motion equation for cells. As is known, the cells in tissues are connected by desmosomes and fit tightly to each other. The cell can begin to move only upon command from the tissue. For example, in the wound-healing process, one observes sudden tissue liquefaction, which occurs under strict control of gene regulation [47]. Also, cancer cells typically have a mesenchymal phenotype and increased motility [45].

In this problem, there is no strict control of cells as it is in living tissue. In this case, each cell looks for a state with a minimum of potential energy, eliminating excessive stretching or compression of the shape. It implies small displacements of the nodes. The movement of any cell is accompanied by significant resistance from the microenvironment, resulting in movement without inertia. Therefore, we can describe the behavior of cells using the approach of Aristotle’s mechanics, in which forces directly determine the velocity of the ξ-th node:

(6) $$v_{x}^{\xi }=\frac{dx^{\xi }}{dt}=kF_{x}^{\xi }H\left(\sqrt{\left({\left(F_{x}^{\xi }\right)}^{2}+{\left(F_{y}^{\xi }\right)}^{2}\right)}-F_{0}\right),$$

(7) $$v_{y}^{\xi }=\frac{dy^{\xi }}{dt}=kF_{y}^{\xi }H\left(\sqrt{\left({\left(F_{x}^{\xi }\right)}^{2}+{\left(F_{y}^{\xi }\right)}^{2}\right)}-F_{0}\right),$$

where *k* is the mobility coefficient and $F_{0}$ is a parameter determining the critical force. H(ζ) stands for the Heaviside step function, H(ζ)=1 at ζ>0, and H(ζ)=0 at ζ⩽0. Equations (6) and (7) contain an additional factor on the right side, implying that the force acts only when it exceeds a certain threshold. This assumption gives additional stability to the cellular environment and prevents excessive fluidity of elements.

The main motor of the system is cell proliferation, depending on environmental conditions. Let us write the space- and time-dependent probability of division of the *i*-th cell in the following form:

(8) $$P_{i}^{\mathrm{div}}\left(t\right)=P_{0}H\left(A_{i}\left(t\right)-mA_{0}\right)T\left(\tau \right)q^{n_{i}\left(t\right)-6},$$

where

(9) $$T\left(\tau \right)=\frac{1}{\sqrt{{\left(1-\tau^{2}\right)}^{2}+\delta \tau^{2}}}.$$

Here, $n_{i}\left(t\right)$ is the current number of nodes of the polygon representing the *i*-th cell; τ stands for the dimensionless shear stress; and $p_{0}$, *q*, and δ are parameters of the problem. The parameter *q* determines the frequency of cell division. In the case of q>1, the more vertices the cell has, the more likely it is that division will occur. As a rule, the cell is either stretched or inflated. We calibrate the distribution (8) so that the most likely shape during evolution would be a hexagonal cell shape, but the emergence of other types of polygons is not excluded. As is known, there are only three types of parquets made from regular polygons: triangular, square, and hexagonal [54]. The most natural is a parquet made of regular hexagons. A hexagon is an energetically favorable form because it is closest to a circle. This conclusion is confirmed experimentally. As was shown in [55], the distribution of cells by the number of sides in the wing epithelium of the *Drosophila* fly looks like a normal one with the most probable number of sides of a cell n=6.

Formula (8) also includes a guard against dividing cells that are too small, as specified by the Heaviside function. The process is initiated only when the cell size exceeds the value $A_{i}\left(t\right)>mA_{0}$. In the work [56], the proliferation process in a zebrafish embryo was studied experimentally. The authors showed that after some cell divides, the total volume of daughter cells is greater than that of the mother cell. At the same time, the size of each daughter cell is smaller than that of the original cell. The authors found that the next division act occurred no earlier than when the volume of the daughter cell reaches approximately 70% of that by the mother cell. Thus, in this work, we fix the value of the parameter m=0.7.

Also, Formula (9) describes the dependence of the cell division probability on the value of the shear stress τ exerted by the liquid on the *i*-th cell. As we noted in the Introduction, the dependence of proliferation on shear stress is resonant: there is a stress value $\tau_{max}$ at which cells divide most intensively. Thus, the function T(τ) takes the following asymptotic values:

(10) $$\lim_{\tau \to 0}T\left(\tau \right)=1,\;\;\lim_{\tau \to \infty }T\left(\tau \right)=0.$$

The first value in (10) means the cell division rate in a liquid at rest. The second one determines the cessation of cell division because of high shear stress.

The resonant value of shear stress $\tau_{res}$ leads to maximum cell growth (δ<2):

(11) $$T\left(\tau_{res}\right)=\frac{2}{\delta \sqrt{\frac{2}{\delta }-1}},$$

where

(12) $$\tau_{res}=\sqrt{1-\frac{\delta }{2}}.$$

Figure 3b schematically illustrates a division mechanism. The algorithm includes the following steps:

1. Determine the longest side of the mother cell.
2. If the number of sides is even, then
  (a) Connect the middle of the longest side with the middle of the opposite side;
  (b) Create two new nodes;
  (c) Remove the mother cell from the list;
  (d) Add two new cells to the list.
3. If the number of sides is odd, then
  (a) Connect the middle of the longest side with the middle of the longest opposite side (choose one of two);
  (b) Create two new nodes;
  (c) Remove the mother cell from the list;
  (d) Add two new cells to the list.

The mitosis process results in a jump in potential energy specified by Equation (2). Elastic energy triggers forces acting on nodes (see Equation (5)). The nodes begin to move, and the volume of new cells grows, gradually reaching the reference value $A_{0}$. Finally, local stress relaxes, and the cellular structure stabilizes. New cells are ready to repeat the cycle.

The mechanism of cell intercalation is necessary to relieve local stresses exerted on the cell in the tissue and to reduce the potential energy of the entire tissue as a whole (Figure 3c). We will assume that the intercalation occurs whenever the bridge between the *i*-th and *j*-th cells becomes less than a critical value $l_{0}$:

(13) $$P_{ij}^{\mathrm{int}}\left(t\right)=H\left(l_{0}-l_{ij}\left(t\right)\right),$$

where H(ζ) is the Heaviside function. Figure 3c schematically shows the intercalation process. As shown in the figure, if the length of the border separating two cells becomes less than $l_{0}$, the segment $l_{24}$ is replaced by a longer segment $l_{13}$ oriented orthogonally. More precisely, we rotate the new side by an angle of π/2 and perform the subsequent extrusion of this side of the polygon by 5% of its original length. As a result, cells #1 and #3 come into direct contact with each other.

On the solid–tissue interface Γ, we set the slip boundary condition for the velocity of the cell nodes:

(14) $$v_{\xi }\cdot {n|}_{\Gamma }=0,$$

where n is the unit vector normal to the boundary (Figure 3a). The slip condition means that the molecules of the cell membrane are essentially sliding along the solid surfaces at a molecular level. This is consistent with the experimental observations of cell behavior.

Equations (1)–(14) provide a complete mathematical formulation to simulate the tissue growth. Generally, it is an individual-based model. It implies that a cell does not lose its individuality and retains information about its evolutionary trajectory.

### 2.2. Hydrodynamic Model for Shear Stresses

**Figure 4:**
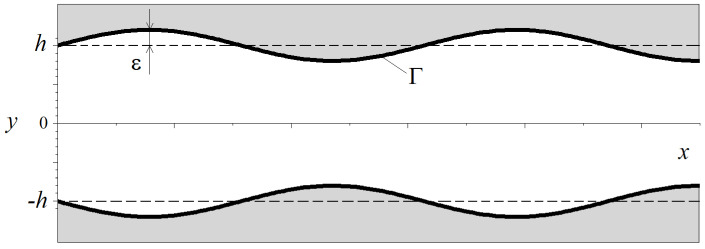
The top view of a sinusoidal wavy channel and coordinate axes.

To simulate cell growth in a perfusion bioreactor, we must know the shear stresses throughout the entire volume of liquid. In this work, we consider flow in a pore of variable cross section. For simplicity, we will consider a 2-D channel with walls that change according to a harmonic law:

(15) $$y_{0}\left(x\right)=\pm h\left(1+\varepsilon \sin\left(Kx\right)\right),$$

where *h* is the channel half-thickness and ε and *K* are the amplitude and wave number of channel variations, respectively. We introduce a Cartesian coordinate system originating in the center of the channel with an *x*-axis directed in the horizontal direction and a vertical *y*-axis directed upward, as shown in Figure 4. The cavity is filled by an incompressible Newtonian liquid characterized by constant dynamic viscosity $\eta_{0}$ and density $\rho_{0}$. Since the presence of the gravitational field in this problem is not essential, we assume that the system is under a weightlessness condition.

The flow of the liquid in a pore channel under a constant pressure difference is governed by the Navier–Stokes equation:

(16) $$\rho_{0}\left(\frac{\partial u}{\partial t}+\left(u\cdot \nabla \right)u\right)=-\nabla p+\eta_{0}\Delta u,$$

which must be supplemented by the boundary condition:

(17) $${u|}_{\Gamma }=0.$$

Here, $u:(u_{x},u_{y})$ stands for the 2-D velocity of the liquid, and *p* is the pressure.

The filtration of the nutrient solution through a scaffold, where the typical pore size ranges from $10^{2}$ to $10^{3}$μ, occurs at low Reynolds numbers. For example, for water flow at typical velocity $u_{0}=10^{-3}$ m/s through channel $h=10^{-4}$ m, we obtain the following estimate:

Re=u0hη0≈0.1.

Consequently, the flow in a porous scaffold is usually laminar. In what follows, we will consider only laminar and stationary fluid flows through the channel. Then, Equation (16) is reduced to the following equation of motion:

(18) $$\nabla p=\eta_{0}\Delta u.$$

In the case of a straight channel (ε=0), a Poiseuille flow is realized in the channel, the velocity field of which has one component: $u:(u_{x}\left(y\right),0)$. Then, one can easily find an analytical solution for problems (17) and (18):

(19) $$u_{x}\left(y\right)=\frac{\Delta p}{4\eta_{0}\Delta x}\left(h^{2}-y^{2}\right),$$

where Δp is the pressure drop applied to a pipe section of length Δx. The Poiseuille flow (19) has a maximum velocity on the axis of symmetry of the pipe and drops to zero at the solid boundaries due to the no-slip condition of a viscous fluid at a solid body.

For this 1-D case, let us introduce the following quantity of the pressure dimension:

(20) $$\tau_{0}=\eta_{0}\frac{du_{x}}{dy},$$

which stands for the shear stress of fluid. Based on (19), one can calculate the shear stress field:

(21) $$\tau_{0}=-\frac{\Delta p}{4\Delta x}y.$$

The shear stress (21) is zero at the center of the channel and takes on a minimum value at Γ. This implies that the fluid element is pulled back because of the boundary condition (17). To determine $\tau_{0}$ on Γ, one needs to change the sign of (21). Therefore, the solid surface element experiences forward thrust (with a positive value). A similar stretching effect is exerted on the cell, depending on where this cell is in the pore.

A general analytical solution for the flow in a 2-D channel with a variable cross section was derived in [57]. We follow this approach and obtain an analytical formula for shear stresses in a wavy channel. By taking into account the incompressibility of the fluid, we introduce a stream function ψ, which satisfies

(22) $$\begin{array}{ccc} & & u_{x}=\frac{\partial \psi }{\partial y},\;\;u_{y}=-\frac{\partial \psi }{\partial x}. \end{array}$$

This allows representing the Stokes Equation (18) in the following form:

(23) ΔΔψ=0.

We look for a solution to Equation (23) with boundary condition (17) in the form of the expansions in powers of ε:

(24) $$\psi \left(x,y\right)=\psi_{0}+\varepsilon \psi_{1}+O\left(\varepsilon^{2}\right),$$

where the first term describes the flow in a straight pipe (ε=0) and the second one corrects it. To satisfy the boundary condition on the wavy walls (see Figure 4), it is convenient to transform the variables x,y into ϑ,φ by

(25) $$\vartheta =\frac{x}{h},\;\varphi =\pm \frac{y}{h(1+\varepsilon \sin(Kx))}.$$

So a wavy channel is transformed into a channel confined by parallel walls φ=±1.

By substituting expansions (24) into (17) and (23) and by collecting terms of the same order of magnitude, we obtain the problems in the first two orders, respectively,

(26) $$\begin{array}{ccc} & & \frac{\partial^{4}\psi_{0}}{\partial \varphi^{4}}=0,\\ \varphi & = & \pm 1:\;\frac{\partial \psi_{0}}{\partial \vartheta }=0,\;\frac{\partial \psi_{0}}{\partial \varphi }=0 \end{array}$$

and

(27) $$\begin{array}{ccc} \frac{\partial^{4}\psi_{0}}{\partial \vartheta^{4}}+2\frac{\partial^{4}\psi_{0}}{\partial \vartheta^{2}\varphi^{2}} & + & \frac{\partial^{4}\psi_{0}}{\partial \varphi^{4}}+12\varphi \frac{\partial^{2}y_{0}}{\partial \vartheta^{2}}+\varphi \left(1-\varphi^{2}\right)\frac{\partial^{4}y_{0}}{\partial \vartheta^{4}}=0,\\ \varphi & = & \pm 1:\;\frac{\partial \psi_{1}}{\partial \vartheta }=0,\;\frac{\partial \psi_{1}}{\partial \varphi }=0. \end{array}$$

By sequentially solving problems (26) and (27), we finally obtain an analytical expression for the dimensionless stream function in (ϑ,φ)-coordinates:

(28) $$\psi_{0}=\varphi -\frac{1}{3}\varphi^{3},$$

(29) $$\psi_{1}=\sin\left(Kd\vartheta \right)\left(2\frac{\cosh(Kh)\sinh(Kh\varphi )-\varphi \sinh(Kh)\cosh(Kh\varphi )}{\cosh(Kh)\sinh(Kh)-Kh}-\varphi +\varphi^{3}\right),$$

where $u_{0}$ is undisturbed fluid velocity at the channel inlet. The velocity field u in (x,y) coordinates can be obtained from (28) and (29) via inverse transformation (25).

The formula for tangential stresses, taking into account the smallness of the amplitude ε≪1, is as follows:

(30) $$\tau \left(x,y\right)=\left(n\cdot \nabla U\right)=\left(\frac{u_{x}u_{y}}{U^{2}}\left(\frac{\partial u_{y}}{\partial y}-\frac{\partial u_{x}}{\partial x}\right)+\frac{u_{x}^{2}}{U^{2}}\frac{\partial u_{x}}{\partial y}-\frac{u_{y}^{2}}{U^{2}}\frac{\partial u_{y}}{\partial x}\right),$$

where n is a unit vector orthogonal to the fluid velocity u, *U* is the velocity magnitude. In (9), the field τ(x,y) is written in the dimensionless form using the value $\tau_{res}$, at which the process of cell growth occurs most intensively.

### 2.3. Numerical Method and Quantitative Measurements

The system of ODEs and the related Formulas (1)–(14), coupled with the equation for the shear stresses (30), was numerically solved using the Euler explicit method, whose stability was warranted by a sufficiently small time step provided by the Courant rule. Usually, we start the simulation from a single cell placed at the entrance to a channel. Table 1 gives the values of model parameters used in all numerical simulations.

The calibration of the parameters was based on the wound-healing problem. Most key parameter values (η, $A_{0}$, $L_{0}$, *q*, $P_{0}$) were taken from the paper by [47], and the benchmark simulation was performed with experimental data presented by [58]. Some parameter values (for example, μ, *k*, $F_{0}$, $l_{0}$) were modified to reflect the properties of cells growing in a scaffold. Here, we used our experience to model cancer cells that usually demonstrate increased motility compared to epithelial cells (see [45]). Finally, some values were chosen empirically so that the mathematical model would be structurally stable and the results would not contradict the general principles of cell tissue development.

To characterize the development of the tissue in the scaffold pore, we apply various measurements during the numerical simulations. The fields A(t,x,y) and L(t,x,y) of cells represented by sets of areas $A_{i}\left(t\right)$ and perimeters $L_{i}\left(t\right)$, respectively, provide information about how cells are distributed in space. These fields can be spatially averaged and normalized by the reference values at successive times

(31) $$A_{1}\left(t\right)=\frac{1}{N\left(t\right)A_{0}}\sum_{i=1}^{N(t)}A_{i}\left(t\right),\;L_{1}\left(t\right)=\frac{1}{N\left(t\right)L_{0}}\sum_{i=1}^{N(t)}L_{i}\left(t\right),$$

to yield ensemble-averaged values of the area and the perimeter, respectively.

A crucial characteristic of the medium is pressure, which helps to identify cells that experience the greatest (or least) load from their microenvironment. We calculate the dimensionless pressure inside the *i*-th cell with an analogy with the pressure in a bubble:

(32) $$p_{i}\left(t\right)=\frac{\sqrt{A_{0}}}{\sqrt{A_{i}\left(t\right)}}-1.$$

One can see that if a free cell is hexagonal, the pressure inside it is zero. When its current area $A_{i}\left(t\right)$ deviates from the reference value $A_{0}$, the pressure either increases ($A_{i}\left(t\right)<A_{0}$) or decreases ($A_{i}\left(t\right)>A_{0}$). By considering the cluster of N(t) cells, one obtains information about the spatial properties of the pressure field p(t,x,y). The instantaneous average value of the pressure is calculated as the arithmetic mean of the pressures in all cells at time *t*:

(33) $$p_{1}\left(t\right)=\frac{1}{N(t)}\sum_{i=1}^{N(t)}p_{i}\left(t\right).$$

The distribution of elastic potential energy E(t,x,y) in space and time is given by Equations (1) and (2). It is beneficial to calculate the instantaneous average energy per cell:

(34) $$E_{1}\left(t\right)=\frac{1}{N(t)}\sum_{i=1}^{N(t)}E_{i}\left(t\right),$$

which characterizes the deformation of tissue developing under the influence of the pore geometry and the shear stresses.

## 3. Numerical Results

### 3.1. Uniform Cell Growth in a Scaffold Pore

**Figure 5:**
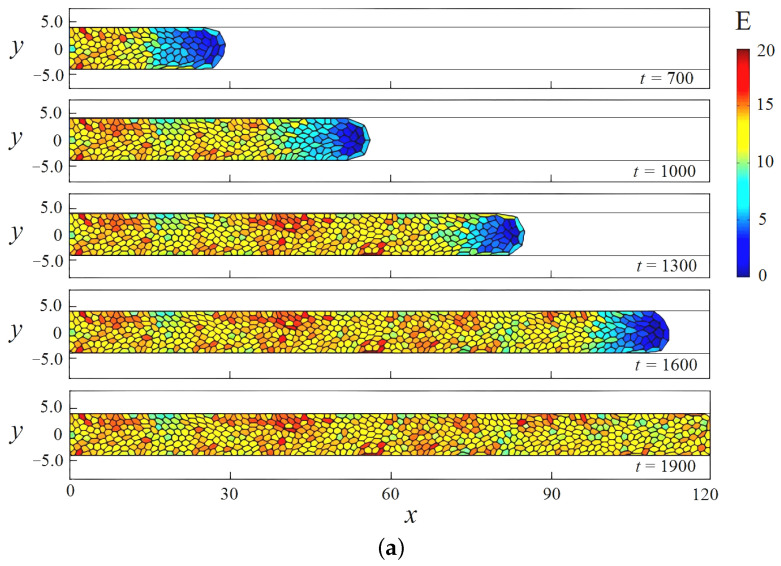

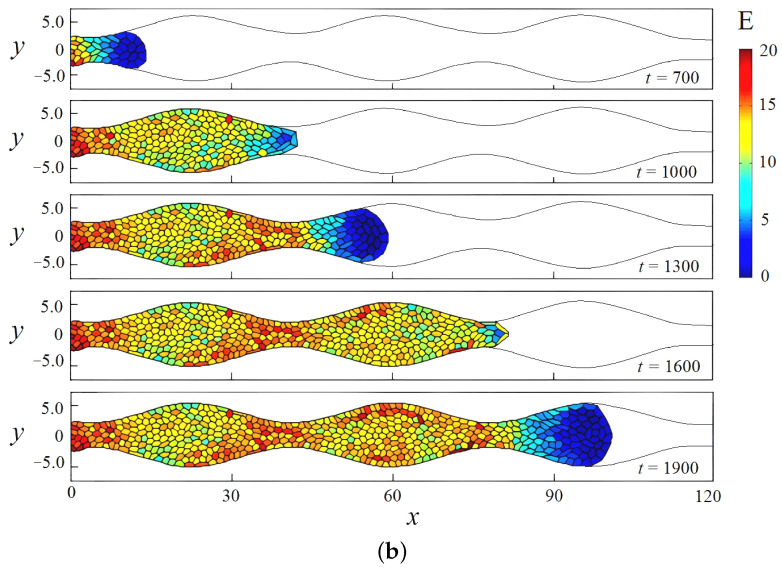
Time evolution of a cell cluster during its growth in straight (**a**) and wavy (**b**) channels. Frames from top to bottom correspond to successive times. The color toolbar shows the distribution of cells in terms of elastic energy. The domain is defined by h=4.5 (**a**) and h=4.5, ε=0.2, K=1/6 (**b**). The effect of shear stresses is not taken into account.

Let us consider the process of the occupation of the scaffold pore with cells for the cases of straight and wavy channels. We assume that both channels have the same volume. We also enable all cells in the cluster to receive enough nutrition for a steady growth at any given time. The latter means that the tissue grows uniformly throughout the occupied space. Figure 5 shows the evolution of the cell cluster at successive times. The domain of integration is defined by

**Figure 6:**
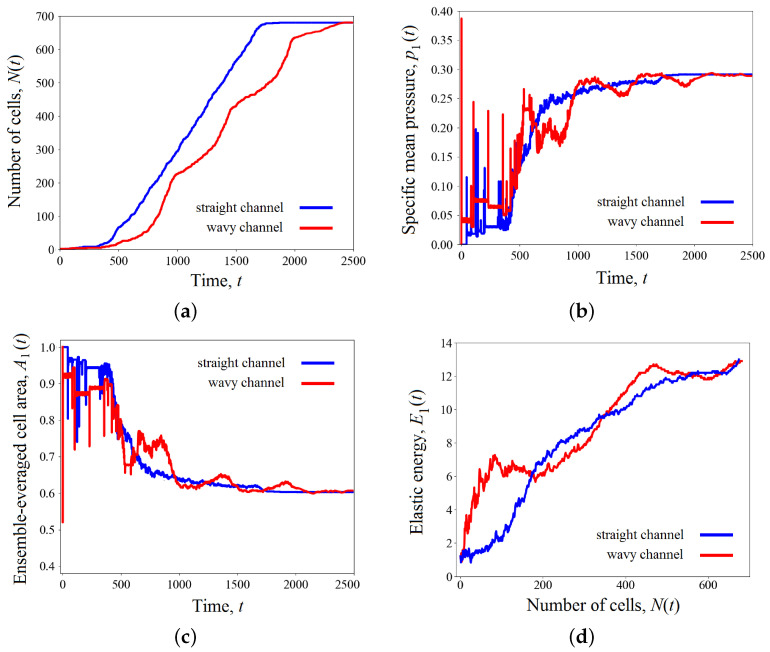
Variations in the total number of cells *N* (**a**), the mean pressure $p_{1}$ (**b**), the average cell area $A_{1}$ (**c**) over time; the specific elastic energy $E_{1}$ (**d**) as a function of cell number. The results were averaged over the ensemble of realizations. The results for straight and wavy channels are marked in blue and red, respectively.

0⩽x⩽120,−4.5⩽y⩽4.5;

0⩽x⩽120,−4.5(1+0.2sin(x/6−π/2))⩽y⩽4.5(1+0.2sin(x/6−π/2))

in the case of straight and wavy channels, respectively. Each frame represents a distribution of cells in terms of elastic energy $E_{i}\left(t\right)$ across the channel space. Also, Figure 6 presents variations in quantitative measurements of the process under study over time: total number of cells N(t) (Figure 6a), the mean pressure $p_{1}\left(t\right)$ (Figure 6b), the average cell area $A_{1}\left(t\right)$ (Figure 6c), and the specific elastic energy $E_{1}\left(t\right)$ (Figure 6d).

As we can see from the figures, the tissue growth in a straight channel is more intense than in a wavy one. For example, at time t=1000, the cell cluster developing in a straight pipe includes approximately 300 cells (Figure 6a), while the number of cells in a wavy channel is significantly smaller—about 200 cells (Figure 6b). This results in the final occupation of the pore volume in a straight channel completed by time t=1700, while the process in a wavy channel lasts longer by 800 time units. It is worth remembering that tissue development in both cases occurs under the same conditions, except for the geometry properties of the channel. This difference seems to be because of the presence of “bottlenecks” in a sinusoidal channel, which conditionally divide it into compartments. The narrowness of the transition between them is controlled by the parameter ε. Our calculations show that for small amplitudes ε≪1, the difference in the development of the cell clusters in a straight and slightly wavy channel becomes insignificant. As the amplitude ε increases, the effect of heterogeneous tissue growth increases. The amplification process is related to ε nonlinearly. Numerical simulations shown in Figure 5 and Figure 6 are performed for ε=0.2, which already has a significant impact on the process of tissue growth. In the case of a wavy channel, the transition of the tissue between the compartments leads to some constriction of the cells, which is expressed by a decrease in their characteristic size (Figure 5) and a local increase in pressure (Figure 6b). The numerical results demonstrate that the tissue in a wavy channel grows through successive shocks (“crises”), which are required to overcome the narrow transition between compartments.

After completion of the transition process, the cells find shapes that are more favorable from an energetic point of view. Since the volumes of the scaffold pores are equal, the ensemble-averaged cell area at the end of both processes takes the value of approximately $0.6A_{0}$ (Figure 6c). Here, strong fluctuations in the average pressure and cell area are observed.

**Figure 7:**
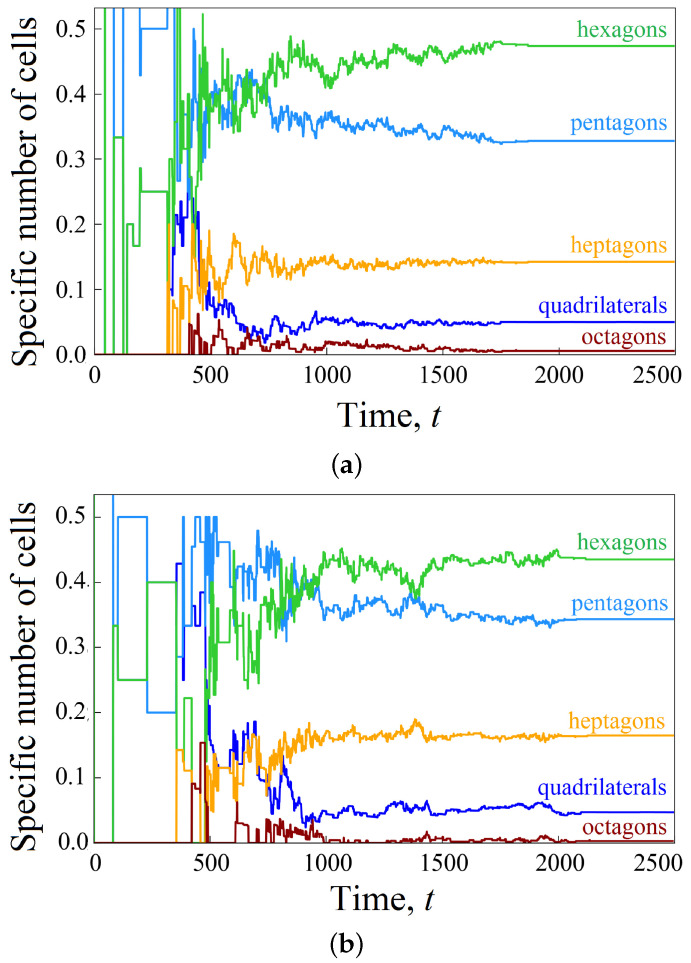
Time evolution of the specific number of cells n/N of a given shape (marked by the different colors) calculated to characterize the development of a cellular cluster in straight (**a**) and wavy (**b**) channels.

Figure 6d shows the dependence of the ensemble-averaged elastic energy of one cell. In a straight channel, one observes an almost uniform increase in potential energy as a scaffold pore fills (Figure 7a). This means that the number of cells grows evenly, as shown in Figure 6a, and the packing density gradually increases, which leads to a redistribution of the crowded cells throughout the pore space. Tissue growth in a wavy channel follows a different scenario. At the beginning of evolution, one can observe a sharp increase in potential energy in the time range 0⩽t⩽800 when the cells overcome the first bottleneck in the pipe (see Figure 5b and Figure 6d). At this stage, the cluster includes no more than 100 cells. Then, tissue growth proceeds at an approximately constant specific energy per cell $E_{1}\approx 6$, which implies the occupation of the new free space of the channel expansion in the first compartment (300⩽t⩽1000). At this stage, the number of cells in the cluster increases rapidly. For example, at time t=1000, there are already more than 250 cells. When the cell mass encounters the next bottleneck at t=1000 (Figure 5b), the specific energy again begins to rise as the tissue is forced to contract (Figure 6d). Then, the process is repeated. Thus, the cluster growth rate changes periodically over time. This fact is also clearly visible when analyzing pressure and average cell area curves (Figure 6b and Figure 6c, respectively).

Helpful information is provided by studying the distribution of cells according to the type of a polygon. Figure 7 shows the time dynamics of the distribution of cells in the cluster according to the number of sides of the polygon. It is worth noting that during the rapid tissue growth, cells with a smaller number of sides predominate in the cluster. For example, at time t=600, the top three most common cell shapes in a wavy channel include pentagons (40%), hexagons (30%), and quadrangles (15%). When the pore space is close to being filled, cells with a larger number of sides predominate, as follows: hexagons (45%), pentagons (35%), and heptagons (20%).

Thus, in this section, we showed that the rate of tissue growth in a straight channel is, on average, higher than in a wavy channel. The mechanism of growth retardation in the second case is associated with the appearance of “bottlenecks”, the intersection of which requires additional energy from the cells. The effect depends nonlinearly on the waviness parameter ε.

### 3.2. Shear Stresses in the Fluid Flow through a Wavy Channel

**Figure 8:**
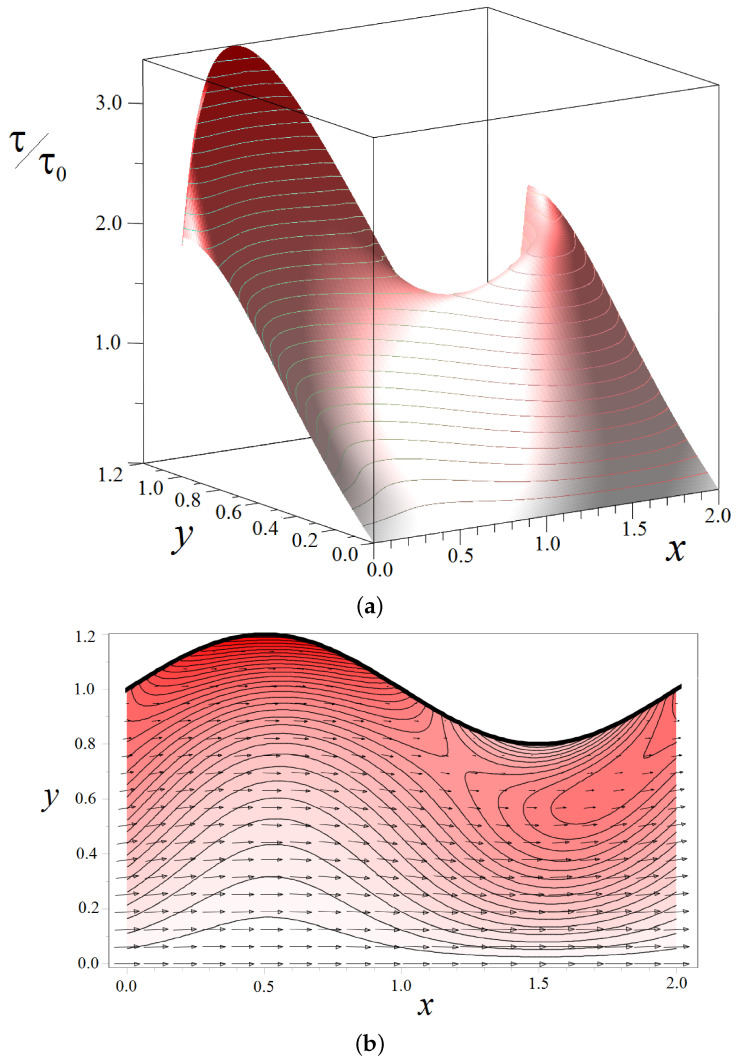
Distribution of the normalized shear stresses $\tau /\tau_{0}$ in a wavy channel characterized by amplitude ε=0.2 and wavenumber K=π: (**a**) 3-D plot with isolines; (**b**) density plot on (*x*,*y*)-plane. The arrows correspond to the fluid velocity. The thick black line shows the channel boundary. Only half of the channel is shown due to the symmetry of the problem.

Let us consider a channel with a half-width h=1. Figure 8 shows the shear stresses, which are calculated using Formulas (22)–(25) and (28)–(30), in a wavy channel with the fixed amplitude ε=0.2. As can be seen from the figure, in contrast to the direct channel, the stress changes nonlinearly along the boundary Γ. In the area where the channel expands, it takes on a maximum value at the boundary and drops in the place where the channel narrows (Figure 8a). At first glance, this seems paradoxical. However, a detailed inspection of the structure of the vector field of fluid velocity and density plot for the stresses shown in Figure 8b explains why this is so. The liquid passes through the bottleneck as a concentrated jet in the center of the channel, which leads to a displacement of the local maximum of shear stresses from the solid boundary. It is worth noting that the peak value of stresses in a wavy channel exceeds those for a straight pipe. The fluid velocity field in a wavy channel becomes inhomogeneous, so the spatial derivatives of this field also become more complicated.

**Figure 9:**
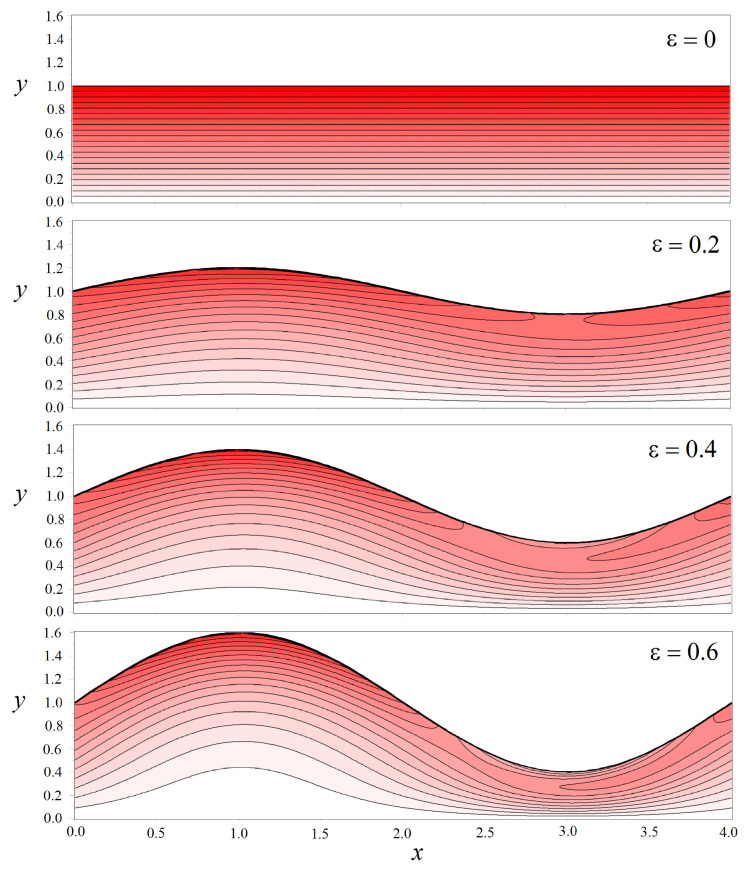
Density plot of the normalized shear stresses $\tau /\tau_{0}$ in the pore space defined by the different amplitudes ε. The wave number is fixed to K=π. The thick black line shows the channel boundary. Only half of the channel is shown due to the symmetry of the problem.

**Figure 10:**
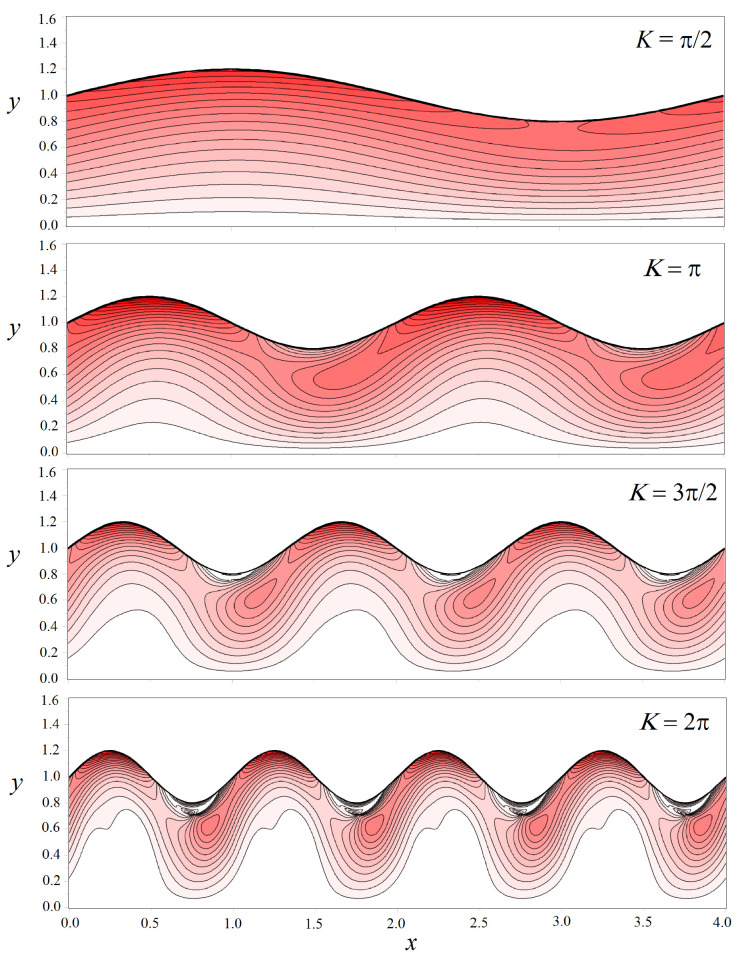
Density plot of the normalized shear stresses $\tau /\tau_{0}$ in the pore space for the different wave numbers *K*. The amplitude is fixed to ε=0.2. The thick black line shows the channel boundary. Only half of the channel is shown due to the symmetry of the problem.

The redistribution of the velocity field in a wavy channel strongly depends on the amplitude ε. Figure 9 shows the density plot of shear stresses in all accessible pore space as the parameter ε varied. The channel wavelength is fixed. Figure 10, on the contrary, shows how the stress field changes depending on the value of the wavenumber *K* at a fixed amplitude ε=0.2.

We can see from Figure 9 and Figure 10 that the field of shear stresses can widely change under the variation in the parameters ε and *K*. In general, in the center of the channel, one can observe the formation of a liquid jet in which the stresses are almost zero. But the values near solid walls increase compared with those calculated for a straight channel (see, for example, Figure 10, K=2π). The peak stress value is reached near the Γ boundary in places of maximal channel expansion.

### 3.3. Cell Growth in a Scaffold Pore Affected by Shear Stresses

Let us consider the influence of shear stresses on the tissue growth in a pore channel. As is known, stress stimulates cells to proliferate. The probability of division can increase several times. However, the effect of shear stresses is resonant. Therefore, there is an optimal value $\tau_{res}$ of the tensile effect, at which it acts most effectively. If the local stress value is greater or less than $\tau_{res}$, then the effect is weakened.

**Figure 11:**
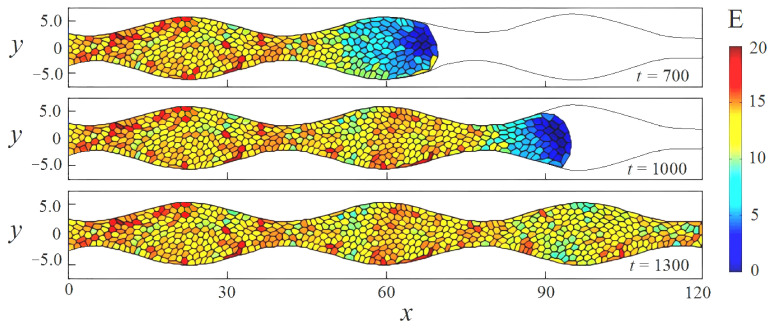
The time evolution of a cell cluster growth is affected by the shear stresses in a wavy channel. Frames from top to bottom correspond to successive times. The color toolbar shows the distribution of cells in terms of elastic energy. The domain is defined by h=4.5, ε=0.2, K=1/6.

**Figure 12:**
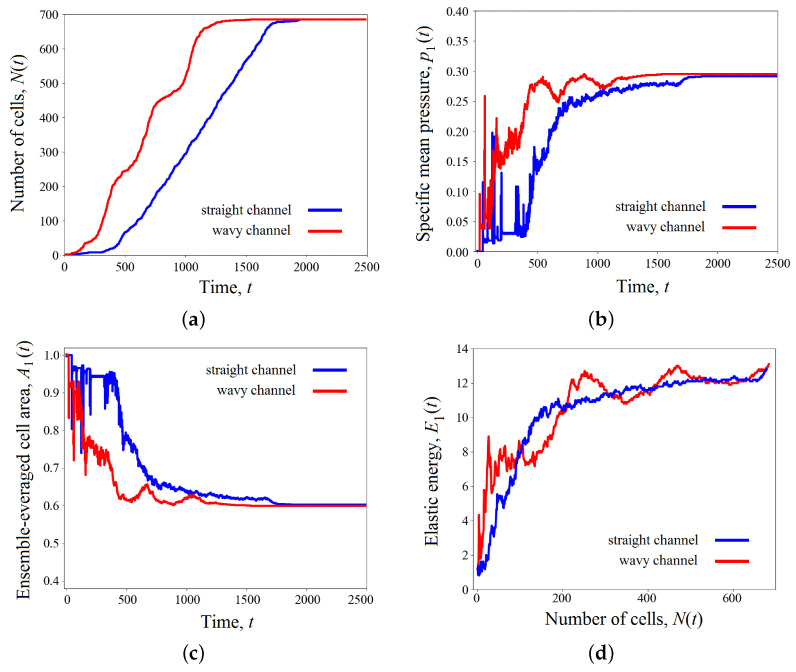
Variations in the total number of cells *N* (**a**), the mean pressure $p_{1}$ (**b**), and the average cell area $A_{1}$ (**c**) for the tissue development affected by the shear stresses; the specific elastic energy $E_{1}$ (**d**) as a function of cell number. The results were averaged over the ensemble of realizations. The results for straight and wavy channels are marked in blue and red, respectively.

**Figure 13:**
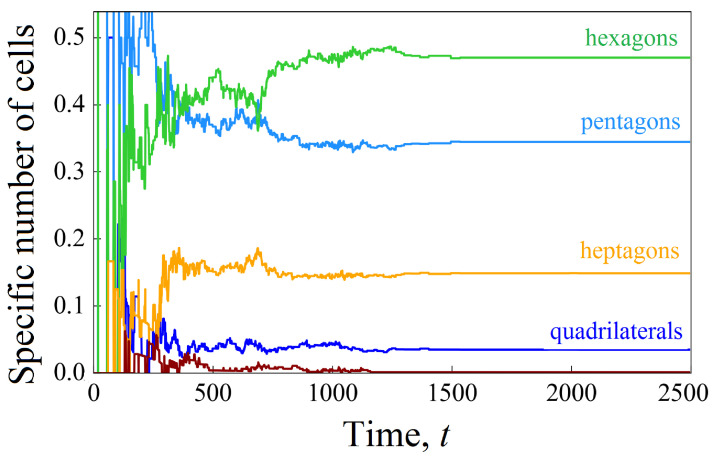
Time evolution of the specific number of cells n/N of a given shape (marked using different colors) calculated to characterize the development of a cellular cluster affected by the shear stresses in a wavy channel.

Figure 11, Figure 12 and Figure 13 present numerical results for the development of a cell cluster in a wavy channel, taking into account the effect of shear stresses. The channel geometry, calculation parameters, and initial conditions are the same as in Figure 6, except taking into account the function T(τ) in Formula (14). As one can see from these figures, the occupation of pore space by cells is accelerated due to the active cell division in new growth zones. These zones are adjacent to the solid boundary in places where the channel widens and, therefore, where the shear stresses reach the peak values shown in Figure 8. These growth zones become generators for the production of new cells. Figure 11 shows that the potential energy increases not only in the narrow places of the channel but also in the places of its greatest broadening. Paradoxically, local jumps in elastic energy in the compartments look even stronger. As the average potential energy of the cluster increases, the cells pass through bottlenecks faster. The filling of the pore space is completed by time t=1200 (Figure 12a). Thus, the development of a cluster in a wavy channel is ahead of a similar process in a straight channel (compare Figure 6a and Figure 12a). It is worth noting that the cells in a straight channel also receive stimulation from the liquid. However, the shear stresses are uniformly distributed here along the solid boundary without reaching the peak value, as in the case of a wavy channel. Thus, cells in the latter receive an advantage based on the sum of two factors—the channel geometry and the structure of the shear stress field. It is interesting to note that the periodic character of the process in the case of a wavy channel is preserved. All characteristics of the cluster oscillate (Figure 12). The dynamics of the specific potential energy appear remarkable as the oscillations become more complex due to the additional contribution of new cell growth zones (compare Figure 6d and Figure 12d).

It is interesting to compare the composition of the cellular cluster (Figure 13). The rapid growth of tissue stimulated by shear stresses leads to the fact that the distribution of cells along the number of sides of the polygon is more reminiscent of the process in a straight channel, as shown in Figure 7a. Hexagons (45%), pentagons (35%), and heptagons (15%) form the top three species. Here, too, at the first stage, pentagons predominate, but as the channel fills, there are more hexagons (compare Figure 7a and Figure 13).

## 4. Discussion and Conclusions

In this work, we numerically study the occupation of scaffold pores by a dynamic cell culture. The scaffold is included in the perfusion bioreactor circuit, which ensures the filtration of the nutrient solution through a porous matrix seeded with cells. To study the system numerically, we apply a vertex model of cell tissue, which makes it possible to consider biomechanical processes in the microenvironment of cells, as well as take into account the influence of shear stresses from the liquid. Such detailed information is helpful when designing a new generation of functional-graded scaffolds that mimic the structure of living tissue.

Our numerical simulations show that, under the same initial conditions, tissue growth in a straight channel of the same volume proceeds faster than in a wavy channel. This is explained by the presence in the latter case of bottlenecks, which significantly delay the development of the cell cluster. As cells pass through such a bottleneck, they are forced, on average, to compress more strongly, which leads to an increase in the potential energy of the tissue and a slowdown in the process of cell division.

We found, however, that in places where the wavy channel thickens, the shear stresses near the solid boundary increase sharply. The stress distribution throughout the space of a pore filled with a moving liquid strongly depends on the amplitude and wavelength of the channel curvature. As is known, such tensile stresses can significantly stimulate cells to divide. In some cases, one could observe an increase in the growth rate of up to one order of magnitude. We show in this work that the peak shear stress not only compensates for the delay in tissue growth, compared with a similar process in a straight channel, but can even lead to faster growth of a cell cluster in a wavy channel. Thus, we can conclude that the manipulation of the internal pore structure of the scaffold can effectively change the rate at which cells occupy it. This finding is important because it provides the basis for programming the scaffold structure to control tissue growth.

In our opinion, this work may be of interest primarily to developers of scaffolds for perfusion bioreactors. In short, we offer the scaffold designer a kind of “microscope” with which they can look in silico into the specific scaffold pore, examine the processes there, and possibly modify the pore microstructure to achieve greater efficiency.

## Figures and Tables

**Table 1:** Values of parameters of a bio-mechanical model of growing tissue.

μ	η	*k*	$F_{0}$	*a*	$L_{0}$	$A_{0}$	$P_{0}$	*q*	*m*	δ	$l_{0}$
1.0	4.0	0.5	0.1	2	6	$3\sqrt{3}/2$	$5\times 10^{-5}$	1.4	0.7	$4\times 10^{-2}$	0.15

## Data Availability

The data that support the findings of this study are available from the corresponding author upon reasonable request.
